# Development of a nomogram for sperm retrieval at microTESE for idiopathic non‐obstructive azoospermia in a multi‐center cohort study

**DOI:** 10.1111/andr.70111

**Published:** 2025-08-29

**Authors:** Giorgio Ivan Russo, Edoardo Pozzi, Fausto Negri, Marco Falcone, Emanuele Zupo, Mirko Preto, Maria Giovanna Asmundo, Sandrine Chamayou, Murat Dursun, Ateş Kadıoğlu, Andrea Salonia

**Affiliations:** ^1^ Department of Urology University of Catania Catania Italy; ^2^ Department of Urology Vita‐Salute San Raffaele University Milan Italy; ^3^ Division of Experimental Oncology/Unit of Urology URI, IRCCS Ospedale San Raffaele Milan Italy; ^4^ Department of Urology A.O.U. Città della Salute e della Scienza, University of Turin Turin Italy; ^5^ Centro HERA ‐ UMR Turin Italy; ^6^ Section of Andrology, Department of Urology, Faculty of Medicine Istanbul University Istanbul Turkey

**Keywords:** infertility, male, microTESE, sperm analysis, spermatozoa

## Abstract

**Background:**

Non‐obstructive azoospermia (NOA) affects approximately 10% of infertile men and represents a major challenge in assisted reproductive technology (ART). A model that includes histological variants could be helpful in predicting sperm retrieval rate (SRR) after microdissection testicular sperm extraction (mTESE) in patients affected by NOA and without genetic abnormalities

**Objectives:**

To develop and validate a predictive nomogram integrating clinical and histopathological variables to estimate SRR in NOA patients undergoing microTESE.

**Material and Methods:**

A multi‐center retrospective/prospective cohort study was conducted between 2022 and 2024, enrolling 333 men diagnosed with NOA across six academic centers. Preoperative data, including age, body mass index (BMI), hormonal profile (follicle‐stimulating hormone [FSH], luteinizing hormone [LH], testosterone), genetic analysis, and ultrasound‐measured testicular volume, were collected. MicroTESE was performed under microscopic guidance with histopathological evaluation. Predictive factors for successful sperm retrieval were analyzed through logistic regression, and a predictive nomogram was constructed and internally validated using bootstrapping techniques. Multi‐variate logistic regression was performed to identify independent variables associated with SRR in patients with available final pathology. Therefore, a predictive nomogram was developed and validated using 1000 bootstrap samples.

**Results:**

The overall SRR was 52.55%. Multi‐variate analysis identified FSH levels (odds ratio [OR]: 0.97; *p* = 0.049), maturation arrest (OR: 0.04; *p* < 0.01), and Sertoli cell‐only syndrome (SCOS; OR: 0.03; *p* < 0.01) as independent predictors of SRR. The predictive nomogram demonstrated good accuracy, with a *C*‐index of 0.75, sensitivity of 73%, specificity of 82%, and overall accuracy of 77% at a cutoff of 0.33.

**Discussion:**

Using preoperative and histology data, we developed a nomogram to predict SR outcomes in patients with NOA undergoing mTESE. Our findings demonstrate that integrating hormonal and histopathological data enhances predictive accuracy of the model, thus providing a valuable tool for preoperative counseling and clinical decision‐making in couples presenting with infertility.

**Conclusion:**

This validated nomogram effectively predicts sperm retrieval outcomes in NOA patients undergoing microTESE, facilitating improved patient counseling, informed clinical decisions, and optimized patient selection. Prospective external validation and further model refinement are recommended to enhance generalizability and clinical applicability.

## 1 INTRODUCTION

Non‐obstructive azoospermia (NOA) affects approximately 10% of infertile men and represents a major challenge in assisted reproductive technology (ART).^1^, ^2^ Microdissection testicular sperm extraction (mTESE) is considered the most adequate surgical approach for sperm retrieval (SR) in men with NOA.^3^However, the success rate of mTESE varies based on individual patient characteristics, including hormonal profile and histopathological findings.^4^, ^5^While some studies have explored predictive markers of SR at mTESE, there is a lack of a reliable, clinically applicable predictive model.^6^ Indeed, SR rate (SRR) after mTESE in men with NOA varies depending on patient characteristics and underlying etiology. Overall, reported SRR ranges from 35% to 60%, with higher success rates in men with conditions such as Klinefelter syndrome compared with idiopathic NOA.^7^ Therefore, a predictive model that could help to better predict SR would be clinically useful by helping to improve reproductive couple's counseling, optimize patient selection, and reduce unnecessary surgical procedures.

Assessing clinical outcomes of TESE in NOA is critical, as the procedure represents the last available option for achieving biological fatherhood in these patients. Beyond SR itself, understanding predictors of pregnancy and live birth after intracytoplasmic sperm injection (ICSI). It is essential for informed counseling and clinical decision‐making. Identifying factors associated with SR and reproductive success can improve patient selection and optimize expectations regarding treatment outcomes.

Interestingly, Liu et al. reported an integrative prediction model for assessing the success rate of SR in men with NOA undergoing mTESE. Researchers analyzed clinical characteristics, hormonal levels, and testicular pathology in 217 patients, identifying key predictive factors such as follicle‐stimulating hormone (FSH) levels, a history of Klinefelter syndrome or cryptorchidism, and testicular histopathology. The model demonstrated strong predictive performance, with an area under the curve (AUC) of 0.781, aiding andrologists in preoperative decision‐making. Patients with relatively normal FSH levels, favorable pathology, and specific medical histories had higher positive SRR.^8^

Of 3093 patients, Ceyhan et al. reported that SR was positive in 1553 (50.2%). Testis volume, chromosome abnormalities, and presence of Y‐chromosome microdeletion and a history of varicocelectomy were shown to significantly affect SRR. A positive SR was higher in men with older age, higher testis volume (>10 mL), lower FSH level (≤12.92 mIU/mL), lower LH level, and higher testosterone level. Only testis volume and patient's age were associated with successful SR at multi‐variate logistic regression analysis^3^.

The etiology of NOA plays a critical role in determining SR success, with conditions like orchitis and cryptorchidism showing markedly higher SRRs compared with idiopathic cases. Understanding the underlying cause not only informs prognosis but also guides individualized counseling and treatment planning for couples pursuing ICSI.

In a study was found to be 39.4% among 627 patients, with variations depending on the etiology; notably, the SRR for orchitis was 90.0%, while for idiopathic cases it was 27.6%^8^.

In fact, based on these premises, a model that includes histological variants could be helpful in predicting SR outcomes after mTESE in patients affected by NOA and without genetic abnormalities. Thereof, we sought to develop and validate a nomogram to predict SR outcomes at mTESE using a multi‐center dataset, integrating key clinical and histopathological variables to enhance decision‐making and patient counseling.

## 2 MATERIAL AND METHODS

### 2.1 Study design and patient selection

This retrospective/prospective cohort study considered data retrieved between 2022 and 2024 across six academic centers. The analyses included men diagnosed with NOA who underwent mTESE for SR and subsequent ARTs. Inclusion criteria were: (1) confirmed NOA based on at least two semen analyses showing azoospermia; (2) preoperative availability of serum FSH, LH, and total testosterone levels; and (3) availability of testicular histopathology findings after surgery. Exclusion criteria included: (1) prior mTESE attempts at other institutions; (2) history of gonadotoxins, for example, chemotherapy or radiotherapy; (3) obstructive azoospermia; and (4) patients with genetic abnormality (karyotype abnormalities; Y‐chromosome microdeletions).

### 2.2 Surgical procedure

Microdissection TESE was performed following the original surgical technique, as developed and popularized by Schlegel et al.^9^ The procedure was performed bilaterally, if necessary. Testicular tissue was sent for each patient for histopathological evaluation.

Following microdissection testicular sperm extraction (microTESE), patients typically undergo follow‐up to assess both surgical recovery and reproductive outcomes. This includes postoperative clinical evaluation for complications (e.g., hematoma, pain, infection), hormonal re‐evaluation if indicated. In cases where spermatozoa were successfully retrieved, coordination with the assisted reproduction team was established to plan ICSI.

### 2.3 Data collection and outcome measures

Preoperative data included age, body mass index (BMI), basic hormonal milieu (i.e., FSH, LH, testosterone), and testicular volume measured by ultrasound. Intraoperative data included SR outcomes according to immediate embryologist’ tissue evaluation and surgical complications. Histopathological findings were classified into Sertoli cell‐only syndrome (SCOS), maturation arrest, or hypospermatogenesis. The primary outcome was SR, defined as the presence of viable spermatozoa suitable for ICSI. Secondary outcomes included predictive factors associated with successful SR at mTESE.

### 2.4 Statistical analysis

Descriptive statistics were used to summarize baseline characteristics. Continuous variables were compared using Student's *t*‐test or Mann–Whitney *U*‐test, while categorical variables were analyzed using chi‐square or Fisher's exact test. Uni‐ and multi‐variate logistic regression was performed to identify independent variables associated with SR in patients with available final pathology. Therefore, a predictive nomogram was developed and validated using 1000 bootstrap samples. Discrimination of the model was quantified using the *C*‐index. Subsequently, the original data were divided into a training set and a validation set and the predictive performance of the nomogram was evaluated in the training set and internally validated via bootstrapping with 1000 resamples.

The associated net benefit of the model was assessed using decision curve analyses.

All analyses were conducted in Stata (Stata Statistical Software, Stata Corp LP), with significance set at *p* < 0.05.

### 2.5 Ethical considerations

This study was approved by the Institutional Review Board of Centro HERA—UMR (Approval No. 2/2023), and all participants provided informed consent upon enrolment. All procedures were conducted following the Declaration of Helsinki guidelines.

## 3 RESULTS

A total of 333 patients have been included. Table 1 depicts the descriptive statistics of the whole cohort of patients. Overall, SRR was 52.55% (175/333), with no statistical differences among centers (*p* = 0.49). Final pathology was available for 270 (81.1%) patients, showing normal tissue in 44 (16.3%), hypospermatogenesis in 42 (15.56%), maturation arrest in 70 (26.9%), and SCOS in 114 (42.2%) patients, respectively. SRR in patients with final pathology was 45.93% (124/270). Supporting Information Figure S1 shows the patients flowchart of the study.

**TABLE 1:** Baseline characteristics of the whole cohort (*N* = 333).

Variables	
Age (years)	36.0 (32.0–40.0)
Body mass index (BMI; kg/m^2^)	26.0 (23.51–28.09)
LH (mUI/L)	7.15 (4.6–11.0)
Follicle‐stimulating hormone (FSH; mUI/L)	15.55 (8.32–24.67)
Total testosterone (ng/mL)	4.1 (3.1–5.14)
Left testis volume (cc)	12.0 (8.0–15.0)
Right testis volume (cc)	12.0 (10.0–16.0)
Smoking, *n* (%)	90 (27.03)

Data are expressed as median (IQR, interquartile range).

Supporting Information Table S1 shows the baseline characteristics of patients according to SRR.

At the univariate logistic regression analysis, FSH (odds ratio [OR]: 0.95 [95% confidence interval [CI] 0.93–0.97; *p* < 0.01]), SCOS (OR: 0.16 [95% CI 0.09–0.28]; *p* < 0.01), maturation arrest (OR: 0.44 (95% CI 0.25–0.78]; *p* < 0.01), and average testicular volume (OR: 1.05 [95% CI 1.00–1.11]; *p* = 0.02) were significantly associated with positive SR, while age and BMI were not.

At multi‐variate logistic regression model, baseline FSH (mUI/L) (OR: 0.97; *p* = 0.049), maturation arrest (OR: 0.04; *p* < 0.01), and SCOS (OR: 0.03; *p* < 0.01) kept their significant association with SSR (Table 2). However, testicular volume was not a significant predictor (OR: 1.66: *p* = 0.47). Hosmer–Lemeshow showed a value of 8.48 (*p* value = 0.97).

**TABLE 2:** Multi‐variate logistic regression analysis of variables associated with sperm retrieval outcomes.

	Model	
Predictors	Odds ratio (95% CI)	*p* value
FSH (mUI/L)	0.97 (0.95–0.99)	0.049
Histology		
Normal (reference)	‐	
Hypospermatogenesis	0.83 (0.20–3.35)	0.79
SCOS	0.03 (0.01–0.10)	<0.01
Maturation arrest	0.04 (0.01–0.10)	<0.01

Abbreviations: CI, confidence interval; FSH, follicle‐stimulating hormone; SCOS, Sertoli cell‐only syndrome.

**FIGURE 1:**
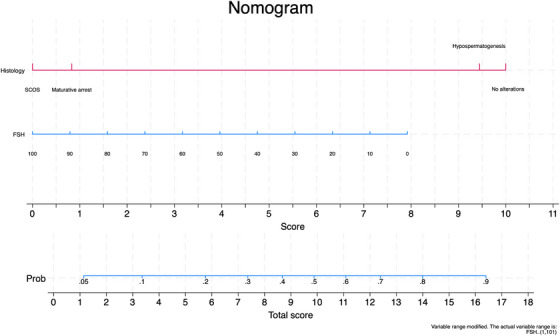
Nomogram predicting the probability of sperm retrieval. Instructions: Draw a line straight upward to the point axis to determine how many points toward the probability of sperm retrieval for each variable. Repeat the process for each additional variable. Sum the points for each of the predictors. Locate the final sum on the total point axis. Draw a line straight down to find the patient's probability of sperm retrieval. FSH, follicle‐stimulating hormone.

Figure 1 graphically illustrates the multi‐variable impact of each variable on the probability of SRR improvement. The model achieved a *C*‐index of 0.75.

**FIGURE 2:**
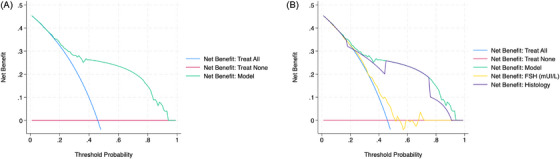
Decision curve analyses demonstrating the net benefit associated with the use of the novel nomogram on sperm retrieval (A) and in comparison, with single predictors (B). FSH, follicle‐stimulating hormone.

The decision curve analysis with the net benefit of the model is shown in Figure 2. Using the 0.33 cut‐off point, of 175 patients 141 (80.57%) were correctly predicted; conversely, 41 (25.95%) cases were misclassified as failures, 34 (19.43%) patients were below the cutoff with SRR and 117 (74.05%) patients above the cutoff without positive SR.

**FIGURE 3:**
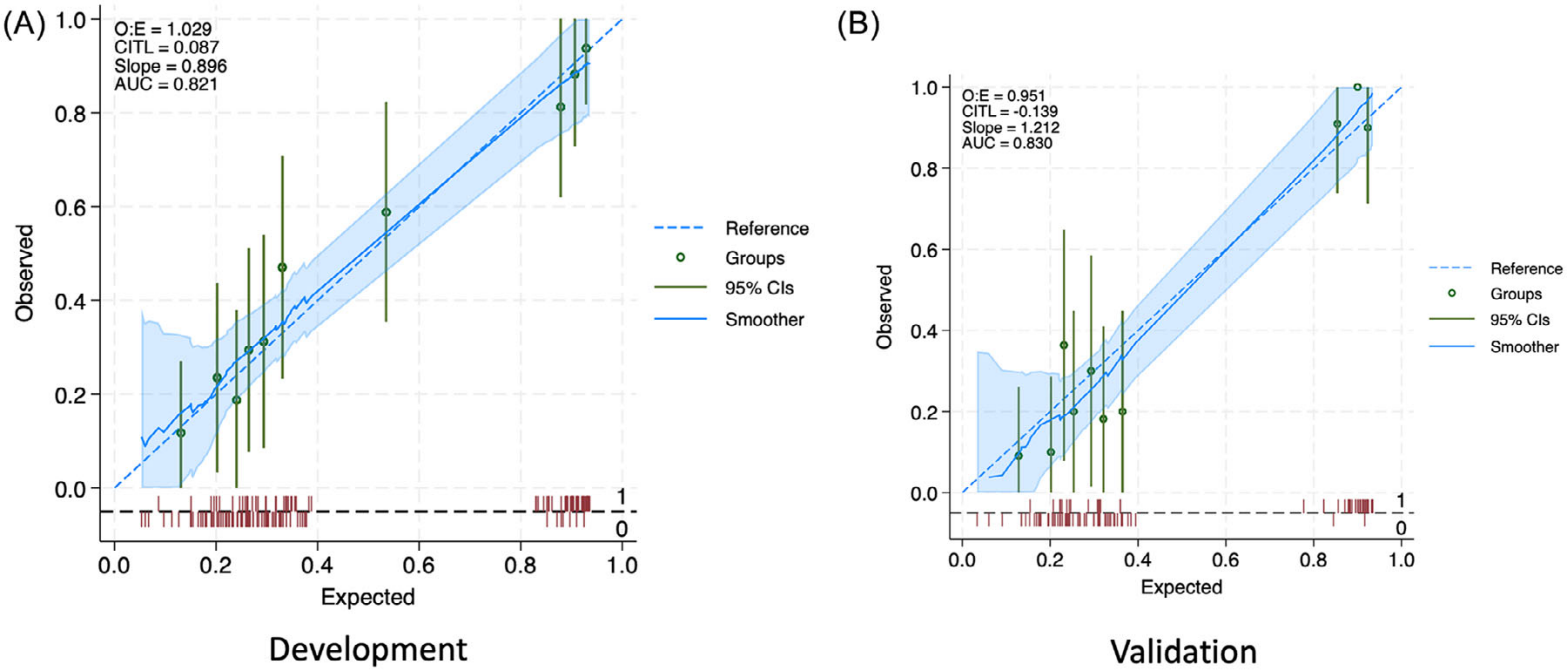
Calibration curve for the nomogram model in the training set (A) and in the validation set (B). AUC, area under the curve; CI, confidence interval.

The model retrieves a sensitivity of 73%, a specificity of 82% with an accuracy of 77% at the cut‐off point of 0.33 (Figure 3).

Figures 3 shows the calibration curve for the nomogram model in the training set (A) and in the validation set (B).

## 4 DISCUSSION

Using preoperative and histology data, we developed a nomogram to predict SR outcomes in patients with NOA undergoing mTESE. Our findings demonstrate that integrating hormonal and histopathological data enhances predictive accuracy of the model, thus providing a valuable tool for preoperative counseling and clinical decision‐making in couples presenting with infertility.

Although clinically not strong enough to dichotomize mTESE outcomes^10^, the significant association between lower FSH levels and improved SRR is consistent with part of the previous literature. Liu et al.^11^ identified FSH as a critical predictor, noting that patients with relatively normal levels had higher SR success rates. However, it should be highlighted that levels of LH and FSH naturally fluctuate throughout the day and are continuously influenced by factors including stress, physical activity, and sleep patterns. Such variability may not reliably reflect the state of spermatogenesis within the testes. Additionally, individual differences in hormonal responsiveness can lead to significant variability in LH and FSH concentrations among men with similar reproductive profiles, complicating the use of these hormones as reliable predictors of micro‐TESE outcomes.

Studies have previously demonstrated that larger testicular volume is associated with higher success rates in SR, likely due to greater preserved spermatogenic tissue^1^, ^2^.However, we did not find such association in the multi‐variate model. We hypothesize that the inclusion of histological results could have added much more positive benefit to the model decreasing the accuracy of testis volume as significant predictor or likely due to collinearity with other covariates or the relatively heterogeneous patient cohort.

Our findings stress the importance of FSH as a potential predictive marker despite not as a stand‐alone variable but in combination with histopathological variants.

Indeed, histopathological characteristics played a pivotal role in our predictive model. In this context, hypospermatogenesis was a strong predictor of successful SR, whereas SCOS and maturation arrest were associated with poorer SR outcomes. These findings align with previous research, where SCOS has been consistently linked to the lowest likelihood of positive SR ^4^. By integrating these histological variants into our model, we further improved its clinical effectiveness.

Recent studies have further explored the relationship between testicular histopathology and SRR. Nguyen et al. emphasized the importance of histological classification in predicting retrieval success, reporting significantly higher SRR in patients with hypospermatogenesis compared with those with SCOS or maturation arrest^12^, ^13^. This supports our model's findings and underscores the importance of histopathological assessment in mTESE planning.

Advancements in imaging technology have also contributed to the prediction of testicular histology in azoospermic patients. Hu et al., for instance, demonstrated that deep learning‐based grayscale ultrasound analysis achieved an AUC of 0.922 in differentiating between normal spermatogenesis and pathologic conditions, such as maturation arrest and SCOS.^6^ The integration of imaging‐based predictive tools with clinical and histopathological data may enhance future SRR models.

It is here of clinical importance to outline that the EAU Guidelines do recommend not to perform any diagnostic invasive investigation (i.e., testis biopsy or fine need aspiration [FNA]) without a therapeutic approach aimed at SR and sperm freezing.^2^

These findings offer meaningful implications for clinical decision‐making in men with NOA undergoing microTESE, even after FNA. The identification of FSH levels, maturation arrest, and SCOS as independent predictors of SR success allows clinicians to better stratify patients based on their likelihood of a positive outcome.

Furthermore, the decision curve analysis demonstrates a favorable net clinical benefit, particularly at the 0.33 probability threshold. In practical terms, this means that patients with a predicted chance of SR below this threshold may be safely spared from undergoing microTESE. This approach can help clinicians reduce unnecessary surgical procedures, lower healthcare costs, and minimize the emotional and psychological burden experienced by couples undergoing fertility treatment.

Overall, the accuracy and clinical utility of our nomogram are highlighted by its robust performance metrics, with a *C*‐index of 0.75, sensitivity of 73%, and specificity of 82%, respectively. Decision curve analysis further validated its net benefit in patient selection. Compared with prior predictive models, our nomogram uniquely incorporates histopathological parameters, providing a more comprehensive risk stratification tool. Conversely, similar to Liu et al., who examined SR outcomes and subsequent pregnancy rates following mTESE and ICSI, highlighting the role of successful SR in improving ART outcomes^7^, our findings further underscore the importance of accurate preoperative predictions to optimize clinical decision‐making and patient expectations.

Although some may doubt about the usefulness of our model, we would like to emphasize how the identification of predictive factors including histology, can draw the way in the future for new therapeutic perspectives. First of all, if we consider patients candidates for redo TESE, knowing the histology can allow better counseling. Second, although not accepted nor practiced, the idea of a potential pre‐surgical testicular biopsy can represent a turning point. In fact, if we take the example of a hypothetical patient who has an FSH of 50, in the absence of pathological alterations, his SRR is 84%, high enough to proceed with surgery.

Despite these strengths, our study has several limitations. First, the retrospective design may introduce selection bias, thus necessitating external validation in a prospective cohort. A key limitation of our study is the absence of external validation. Although the model was internally validated using bootstrapping, its generalizability to other populations remains to be confirmed, in accordance with TRIPOD recommendations. Additionally, inter‐surgeon and inter‐center variability may have contributed to heterogeneity in surgical performance and outcomes, potentially influencing the predictive accuracy of the model. This variability reflects real‐world clinical practice but should be considered when interpreting the results. Second, due to not homogenous availability among centers, we did not add anti‐Mullerian hormone values, which have been discovered to be highly associated with SR outcomes at surgery^14^, ^15^ or inhibin‐B. A key limitation of our study is the absence of data on pregnancy and live birth outcomes following SR and subsequent ICSI. As a result, we were unable to assess the clinical effectiveness of the retrieved spermatozoa in achieving reproductive success, which limits the translational value of our findings for patient counseling and decision‐making. Third, while current analysis has been performed by homogenously excluding men with genetic abnormalities, future studies should explore the integration of genetic markers to enhance model performance.^16^, ^17^, ^18^ Fourth, while mTESE remains the gold‐standard technique for men with NOA, differences in surgical expertise and laboratory processing may affect outcomes across different centers. Standardized protocols should be implemented to mitigate these discrepancies. Fifth, future research should focus on refining predictive models through machine learning approaches, incorporating large‐scale, multi‐center datasets to improve generalizability.

## 5 CONCLUSIONS

We developed a predictive nomogram for SRR at mTESE in infertile men with NOA. By integrating basic preoperative hormonal values and histopathological variables, the model provides a clinically applicable tool for optimizing patient selection and infertile couple's counseling. Further external validation and prospective studies are warranted to refine its predictive accuracy and broaden its applicability in diverse populations.

## AUTHOR CONTRIBUTIONS

*Conceptualization*: Giorgio Ivan Russo, Edoardo Pozzi. *Data curation*: All the authors. *Formal analysis*: Giorgio Ivan Russo. *Investigation*: All the authors. *Methodology*: Giorgio Ivan Russo. *Supervision*: Giorgio Ivan Russo. *Validation*: All the authors. *Writing—original draft*: Giorgio Ivan Russo, Edoardo Pozzi. *Writing—review & editing*: Giorgio Ivan Russo, Edoardo Pozzi.

## CONFLICT OF INTEREST STATEMENT

The authors declare no conflicts of interest.

## Supporting information



Supporting Information

Supporting Information

## Data Availability

Data will be shared upon request to the corresponding author.
