# Supplementary material for: Development of a nomogram for sperm retrieval at microTESE for idiopathic non‐obstructive azoospermia in a multi‐center cohort study
Source: Andrology. 2025 Aug 29;14(5):1202–8. doi: 10.1111/andr.70111 (PMC13266452; doi:10.1111/andr.70111)
Supplement: Supplementary file 1 — Supporting Information [file ANDR-14-1202-s002.doc]

Suppl. Figure 1. Flow chart for the study population

Assessed for eligibility

(n = 333)

Excluded (n = 0)

Final cohort (n = 333)

#

**Analysis**

Assessed for general statistics (n = 333)

Analyzed (n = …)

Excluded from analysis

(n = …) (give reasons)

Analyzed (n = …)

Excluded from analysis

(n = …) (give reasons)

Excluded (n = 63)

- Missing histology (n = 63

Assessed for nomogram (n = 270)
