# Supplementary material for: Development of a nomogram for sperm retrieval at microTESE for idiopathic non‐obstructive azoospermia in a multi‐center cohort study
Source: Andrology. 2025 Aug 29;14(5):1202–8. doi: 10.1111/andr.70111 (PMC13266452; doi:10.1111/andr.70111)
Supplement: Supplementary file 2 — Supporting Information [file ANDR-14-1202-s001.docx]

| **Suppl. table 1. Baseline characteristics of patients according to sperm retrival rate (SRR)** | | | |
| --- | --- | --- | --- |
| **Variable** | **Negative SRR (n= 146)** | **Positive SRR (n= 124)** | **P-value** |
| **Age (years), median (IQR)** | 36 (32–39) | 37 (32–40) | 0.33^†^ |
| **BMI (kg/m²), median (IQR)** | 25.5 (23.5–29.2) | 26.1 (23.4–28.1) | 0.68^†^ |
| **FSH (mUI/L), median (IQR)** | 18.3 (12.8–29) | 12.7 (6.1–20) | <0.01^†^ |
| **Histology** |  |  | <0.01^*^ |
| **Normal** | 4 (2.74%) | 40 (32.26%) |  |
| **Hypospermatogenesis** | 5 (3.42%) | 37 (29.84%) |  |
| **Maturation arrest** | 48 (32.88%) | 22 (17.74%) |  |
| **SCOS** | 89 (60.96%) | 25 (20.16%) |  |
| IQR = interquartile range; BMI = body mass index; FSH = follicle stimulating hormone; SCOS = Sertoli cell only syndrome  † at Mann-Whitney U- test  * at chi-square test | | | |
